# Simulation of Stomatal Conductance and Water Use Efficiency of Tomato Leaves Exposed to Different Irrigation Regimes and Air CO_2_ Concentrations by a Modified “Ball-Berry” Model

**DOI:** 10.3389/fpls.2018.00445

**Published:** 2018-04-09

**Authors:** Zhenhua Wei, Taisheng Du, Xiangnan Li, Liang Fang, Fulai Liu

**Affiliations:** ^1^Center for Agricultural Water Research in China, China Agricultural University, Beijing, China; ^2^Department of Plant and Environmental Sciences, Faculty of Science, University of Copenhagen, Taastrup, Denmark; ^3^Northeast Institute of Geography and Agroecology, Chinese Academy of Sciences, Changchun, China

**Keywords:** CO_2_, alternative partial root-zone irrigation, model simulation, stomatal conductance, water use efficiency, tomato

## Abstract

Stomatal conductance (*g*_s_) and water use efficiency (*WUE*) of tomato leaves exposed to different irrigation regimes and at ambient CO_2_ (*a*[CO_2_], 400 ppm) and elevated CO_2_ (*e*[CO_2_], 800 ppm) environments were simulated using the “Ball-Berry” model (BB-model). Data obtained from a preliminary experiment (Exp. I) was used for model parameterization, where measurements of leaf gas exchange of potted tomatoes were done during progressive soil drying for 5 days. The measured photosynthetic rate (*P*_n_) was used as an input for the model. Considering the effect of soil water deficits on *g*_s_, an equation modifying the slope (*m*) based on the mean soil water potential (Ψ_s_) in the whole root zone was introduced. Compared to the original BB-model, the modified model showed greater predictability for both *g*_s_ and *WUE* of tomato leaves at each [CO_2_] growth environment. The models were further validated with data obtained from an independent experiment (Exp. II) where plants were subjected to three irrigation regimes: full irrigation (FI), deficit irrigation (DI), and alternative partial root-zone irrigation (PRI) for 40 days at both *a*[CO_2_] and *e*[CO_2_] environment. The simulation results indicated that *g*_s_ was independently acclimated to *e*[CO_2_] from *P*_n_. The modified BB-model performed better in estimating *g*_s_ and *WUE*, especially for PRI strategy at both [CO_2_] environments. A greater *WUE* could be seen in plants grown under *e*[CO_2_] associated with PRI regime. Conclusively, the modified BB-model was capable of predicting *g*_s_ and *WUE* of tomato leaves in various irrigation regimes at both *a*[CO_2_] and *e*[CO_2_] environments. This study could provide valuable information for better predicting plant *WUE* adapted to the future water-limited and CO_2_ enriched environment.

## Introduction

Plant stomata aperture play a predominant role in modulating the diffusion of CO_2_ and H_2_O vapor between leaf and atmosphere (Buckley and Mott, 2013), and optimizing photosynthetic and transpiration rates, hereby the water use efficiency (*WUE*) at leaf scale (Liu et al., 2009). It is well-established that both reduced irrigation regimes, especially alternate partial root-zone irrigation (PRI) and elevated atmospheric CO_2_ environment (*e*[CO_2_]) could induce partial stomatal closure and synergistically enhance *WUE* (Pazzagli et al., 2016). Therefore, a better understanding of how to model stomatal conductance for water vapor (*g*_s_) is essential for the accurate prediction of leaf transpiration and improvement of plant *WUE* in response to the future water limited and CO_2_ enriched environments.

A number of approaches has been tested for modeling *g*_s_ under well-watered conditions (Gutschick and Simonneau, 2002). Among those, the Ball-Berry model (BB-model) describing the linear coupling relation of *g*_s_ to photosynthetic rate (*P*_n_), relative humidity (*h*_s_), and CO_2_ concentration (*C*_s_) on the leaf surface (Ball et al., 1987) has been broadly adopted and utilized from leaf to plant scale due to its apparent accuracy and simplicity (Miner et al., 2017). However, the *P*_n_-*g*_s_ relationship of BB-model would be changed under water stress (Damour et al., 2010), hence modified model is needed for simulating *g*_s_, for instance, by incorporating empirical functions coupling with abscisic acid (ABA), leaf water potential or soil water potential (Sala and Tenhunen, 1996; Gutschick and Simonneau, 2002; Bauerle et al., 2004; Damour et al., 2010).

For drought-prone areas, more efficient irrigation techniques need to be developed and implemented in order to achieve optimal crop yield and quality (Du et al., 2015). PRI strategy has been demonstrated to save considerable amount of irrigation water without significantly reducing yield as compared to full irrigation (FI) (Kang and Zhang, 2004; Wei et al., 2016). It is well-known that reduced plant water consumption under PRI is resulted from a decreased *g*_s_, which is primarily regulated by the root-to-shoot ABA signaling triggered in the roots exposed to drying soil (Davies et al., 2002; Liu et al., 2006). A modified BB-model based on the temporal and spatial change of soil water potential (Ψ_s_) in the soil columns has been reported to be capable of predicting *g*_s_ and *WUE* of PRI treated potato leaves, indicating an enhancement of *WUE* for PRI in relation to FI plants (Liu et al., 2009).

Plants grown at *e*[CO_2_] generally possess an increased *P*_n_ but decreased *g*_s_, resulting in a greater *WUE* as compared to those growth at *a*[CO_2_] (Pazzagli et al., 2016). An better understanding of the coordination between *g*_s_ and *P*_n_ in response to *e*[CO_2_] is crucial for simulating the *WUE* at leaf level. The magnitude of *e*[CO_2_] effect on *g*_s_ is modulated substantially together with other environmental variables, if *g*_s_ is independently acclimated to *e*[CO_2_] from *P*_n_, this would alter the sensitivity of *g*_s_ to [CO_2_], *P*_n_, and/or *h*_s_, and thereby requiring re-parameterization of the BB-model for plants grown under different CO_2_ environment (Ainsworth and Rogers, 2007). As lower *g*_s_ and higher *WUE* are anticipated under both *e*[CO_2_] and reduced irrigation strategies, and a synergic interaction of those two factors would further decrease *g*_s_ and enhance *WUE* (da Silva et al., 2017). However, this will complicate the influence of *e*[CO_2_] associated with PRI regime on the prediction of *g*_s_ and *WUE* using the BB-model.

To date, the change of *g*_s_ and *WUE* of tomato leaves in response to PRI strategy at *e*[CO_2_] have not been depicted by any model. Therefore, the objective of this study was to examine whether *g*_s_ is independently acclimated to *e*[CO_2_] and if the BB-model is capable of predicting leaf *g*_s_ and *WUE* of tomato leaves exposed to different irrigation regimes in combination with two CO_2_ growth conditions. Such modeled *g*_s_ might provide an effective way for estimating transpiration rate at canopy scale and optimizing *WUE* of tomato plant in the future drier and CO_2_ enriched environment.

## Materials and methods

### The BB-model and its modification

The BB-model (Ball et al., 1987) describes the relationship between leaf stomatal conductance (*g*_s_) and photosynthetic rate (*P*_n_), relative humidity (*h*_s_) and CO_2_ concentration (*C*_s_) on the leaf surface:

(1) $$\begin{array}{r} g_{s}=mP_{n}\frac{h_{s}}{C_{s}}+g_{0} \end{array}$$

where *g*_0_ is the residual stomatal conductance if *P*_n_ is zero, *m* is the slope of relation between *g*_s_ and *P*_n_*h*_s_/*C*_s_ (the Ball-index), also called the stomatal sensitivity factor. Under well-watered condition, the *m* is a constant and this model is a simple linear correlation between *g*_s_ and Ball-index. However, under soil water deficits, *m* could be varied largely, and the relationship between *g*_s_ and *P*_n_*h*_s_/*C*_s_ becomes curvilinear (Sala and Tenhunen, 1996). Accounting for the effect of soil water deficits on leaf *g*_s_, numerous approaches have been used to adjust the *m*. The modified *m* could be based on an exponential function related to the ABA concentration in the xylem sap (Gutschick and Simonneau, 2002). Our earlier studies found that plant ABA could be empirically expressed as a linear function of the mean soil water potential (Ψ_s_) in the root zone (Liu et al., 2008). Hereby, in this study, the xylem sap ABA was replaced by Ψ_s_:

(2) $$\begin{array}{r} m=m_{i}e^{-\beta \psi_{s}} \end{array}$$

where *m*_i_ is the initial slope of the BB-model without soil water deficits, β is a constant.

In Equation (1), *C*_s_ was calculated as:

(3) $$\begin{array}{r} C_{s}=C_{a}-P_{n}\frac{1.37}{g_{b}} \end{array}$$

where *C*_a_ is the atmospheric CO_2_ concentration (i.e., 400 or 800 ppm in this study); *g*_b_ is the boundary layer conductance and shown to be 9.29 mol m^−2^ s^−1^ in the leaf chamber according to the manufacture's directions. The *h*_s_ is computed as the ratio of two partial pressures of water vapor at the leaf surface and in the leaf internal space of stomata, *e*_s_/*e*_i_. The *e*_s_ was obtained by Equation (4) as shown by Gutschick and Simonneau (2002):

(4) $$\begin{array}{r} {\text{g}}_{\text{s}}\left({\text{e}}_{\text{i}}-{\text{e}}_{\text{s}}\right)={\text{g}}_{\text{b}}\left({\text{e}}_{\text{s}}-{\text{e}}_{\text{a}}\right) \end{array}$$

By rearranging Equation (4), *h*_s_ was calculated as:

(5) $$\begin{array}{r} h_{s}=\frac{e_{s}}{e_{i}}=\frac{\left(e_{a}/e_{i}+g_{s}/g_{b}\right)}{\left(1+g_{s}/g_{b}\right)} \end{array}$$

where *e*_a_ is the partial pressure of water vapor in the air and is obtained during gas exchange measurement. *e*_i_ could be computed by Equation (5) from the leaf temperature (*T*, °C).

(6) $$\begin{array}{r} e_{i}=6.11e^{\left(\frac{7.5\ln\;\left(10\right)T}{T+237.3}\right)} \end{array}$$

It is necessary to notice that *g*_s_ is used as an input variable for computing *h*_s_ (Equation 5). The *g*_s_ could be obtained by rearranging the BB-model as:

(7) $$\begin{array}{r} g_{s}=\frac{mP_{n}\left(e_{a}/e_{i}+g_{s}/g_{b}\right)}{C_{s}\left(1+g_{s}/g_{b}\right)}+g_{0} \end{array}$$

Equation (7) could then be elaborated as a quadratic equation of *g*_s_:

(8) $$\begin{array}{r} \left(C_{s}/gb\right)g_{s}^{2}+\left(C_{s}-g_{0}C_{s}/g_{b}-mP_{n}/g_{b}\right)g_{s}+\left(-\left(g_{0}C_{s}+mP_{n}e_{a}/e_{i}\right)\right)=0 \end{array}$$

*g*_s_ was solved as:

(9) $$\begin{array}{r} g_{s}=\frac{-B+\sqrt{{\left(B\right)}^{2}-4AC}}{2A} \end{array}$$

where A = (*C*_s_/*g*_b_), B = (*C*_s_-*g*_0_*C*_s_/*g*_b_-*mP*_n_/*g*_b_) and C = −(*g*_0_*C*_s_+*mP*_n_*e*_a_/*e*_i_). By applying PROC NLIN (SAS 9.4 Ins. Inc.) of *g*_s_ on the remaining variables, the parameters *g*_0_ and *m* were derived. Here, *m* could be replaced by Equation (2) to consider the effect of soil water deficits on *g*_s_.

After estimating *g*_s_ by Equation (9), leaf transpiration rate (*T*_r_) could be calculated as:

(10) $$\begin{array}{r} T_{r}=\frac{\left(e_{i}-e_{a}\right)}{P_{a}\left(1/g_{s}+1/g_{b}\right)} \end{array}$$

where *P*_a_ is the air pressure (1013 hPa). *WUE* of tomato leaves was then computed as:

(11) $$\begin{array}{r} WUE=\frac{P_{n}}{T_{r}}=\frac{P_{n}P_{a}\left(1/g_{s}+1/g_{b}\right)}{\left(e_{i}-e_{a}\right)} \end{array}$$

The aim of this study was to examine the capability of the BB-model in predicting *g*_s_ and *WUE* for tomato leaves at *e*[CO_2_] in combination with different irrigation regimes as this has not been done up to date; therefore, we have taken the observed *P*_n_ as an input for the BB-model rather than developing a coupled model for *P*_n_. Moreover, we compared the performance of the original BB-model (without *m* modification) and the modified BB-model (with *m* modification by Equation 2) in simulating *g*_s_ and *WUE* in order to evaluate the importance of soil water deficits on the model performance under different irrigation regimes and CO_2_ enriched environment.

### Data

**Figure 1:**
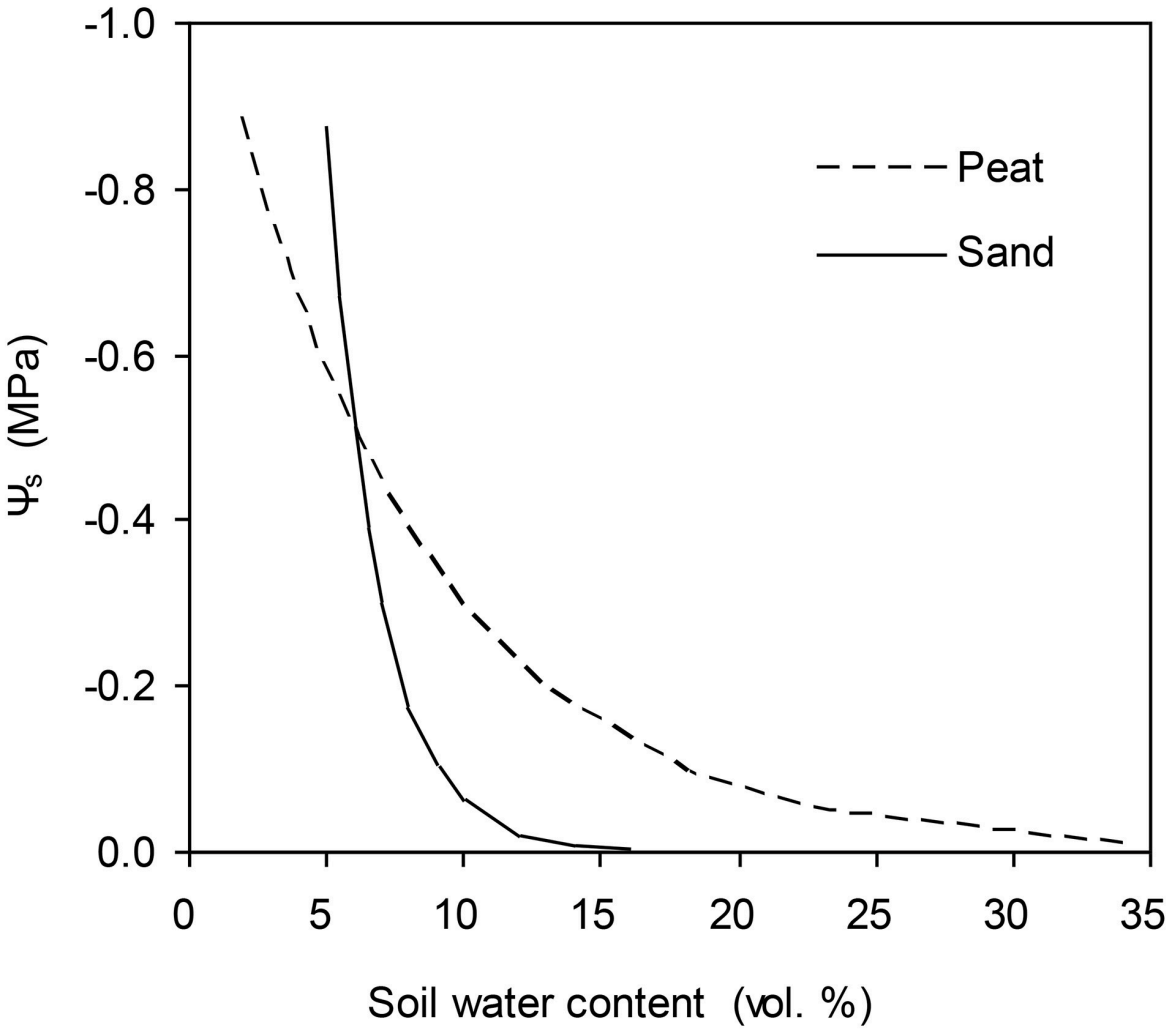
Water retention curves of the peat substrate used in experiment one and of the sandy soil used in experiment two.

The data for this study are from two pot experiments conducted in a climate-controlled greenhouse at the experimental farm of the Faculty of Science, University of Copenhagen, Taastrup, Denmark. The experimental setups have been detailed elsewhere (Yan et al., 2017; Wei et al., 2018) and are only summarized here. In both experiments, the tomato seeds (*Solanum lycopersicum* L., cv. Elin) were sown on 26th Sept. 2016. Half of the plants were grown in a greenhouse cell with ambient CO_2_ concentration of 400 ppm (*a*[CO_2_]), and another half were grown in a cell with elevated CO_2_ concentration of 800 ppm (*e*[CO_2_]). In the first experiment (Exp. I), plants were grown in 1.5 L pots filled with peat substance. Since 31st Oct., plants were subjected to progressive soil drying by withholding irrigation from the pots for 5 days when the *g*_s_ decreased to ca. 10% of that on 31st Oct. (i.e., when pot weight ca. 320 g). Gas exchange measurements (*P*_n_ and *g*_s_) were made at midday around 10:00 h with a portable photosynthetic system (LiCor-6400XT, LI-Cor, NE, USA). Measurements were performed on one leaf per plant at 20°C chamber temperature and 1200 μmol m^−2^ s^−1^ photon flux density, and at a [CO_2_] of 400 ppm for *a*[CO_2_] and 800 ppm for *e*[CO_2_] treatment, respectively. The mean volumetric soil water content in the pots was monitored by weighing the pots daily. The mean Ψ_s_ was then obtained based on the water retention curve for the peat substance (Figure 1). Other environmental variables such as *e*_a_ and *T* were also obtained during gas exchange measurements. The data obtained from this experiment was used for parameterization.

The models were then validated by data obtained from another experiment (Exp. II). In this experiment, tomato plants were grown in pots with roots divided into two equal compartments. The pots (10 L) were filled with a sandy soil. The water retention curve of the sandy soil is shown in Figure 1. Three weeks after transplanting, plants were subjected to three irrigation regimes: (1) full irrigation (FI), in which both soil columns were irrigated daily to 18% (vol.); (2) alternative partial root-zone drying (PRI), in which only one soil column was watered daily to 70% irrigation amount of FI while the other was allowed to dry until the soil water content had decreased to ca. 6%; then the irrigation was shifted; (3) deficit irrigation (DI), in which the same amount of water for PRI was irrigated evenly to the two soil columns. The irrigation treatments lasted 40 days and each soil compartment of the PRI plants had experienced five drying/wetting cycles. The mean soil water content of each soil column was determined by TDR (Time Domain R ctometry; TRASE, Soil Moisture Equipment Corp., USA). The *P*_n_ and *g*_s_ and other environmental variables were obtained in the same way as for experiment I.

### Statistics

The performance of the original and the modified BB-model was compared by evaluating the coefficient of determination (*r*^2^), the mean absolute error (MAE) and the root mean square of error (RMSE) of the linear regressions between the measured and the observed values of *g*_s_ and *WUE*. Analysis of covariance (ANCOVA) (SAS 9.4 Ins. Inc.) was performed to reveal the regression lines between vapor pressure deficit (*VPD*) in the atmosphere and leaf *WUE*.

## Results

### Model parameterization

The data from experiment I was used for model parameterization. In brief, during the 5 days of soil drying Ψ_s_ decreased from −0.01 to −0.53 MPa and −0.01 to −0.61 MPa, *g*_s_ decreased from 0.61 to 0.03 mol m^−2^ s^−1^ and 0.39 to 0.03 mol m^−2^ s^−1^, and *P*_n_ decreased from 15.3 to 1.54 mmol m^−2^ s^−1^ and 18.3 to 3.77 mmol m^−2^ s^−1^, for the plants grown at [CO_2_] concentration of 400 and 800 ppm, respectively.

**Figure 2:**
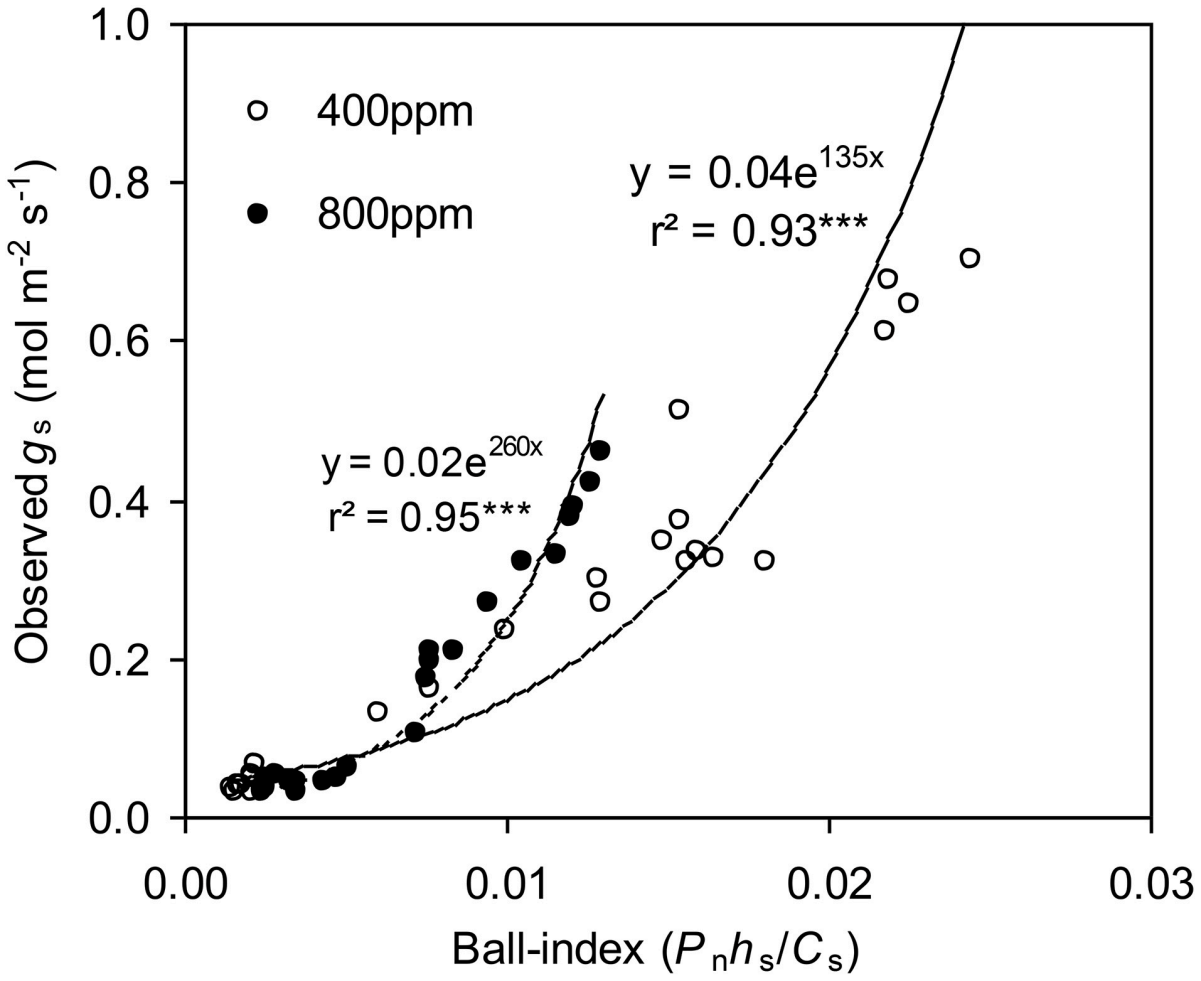
Relationship between the observed stomatal conductance (*g*_s_) and Ball-index (*P*_n_*h*_s_/*C*_s_) of a tomato leaf during progressive soil drying at [CO_2_] concentration of 400 and 800 ppm, respectively in experiment one.

**Figure 3:**
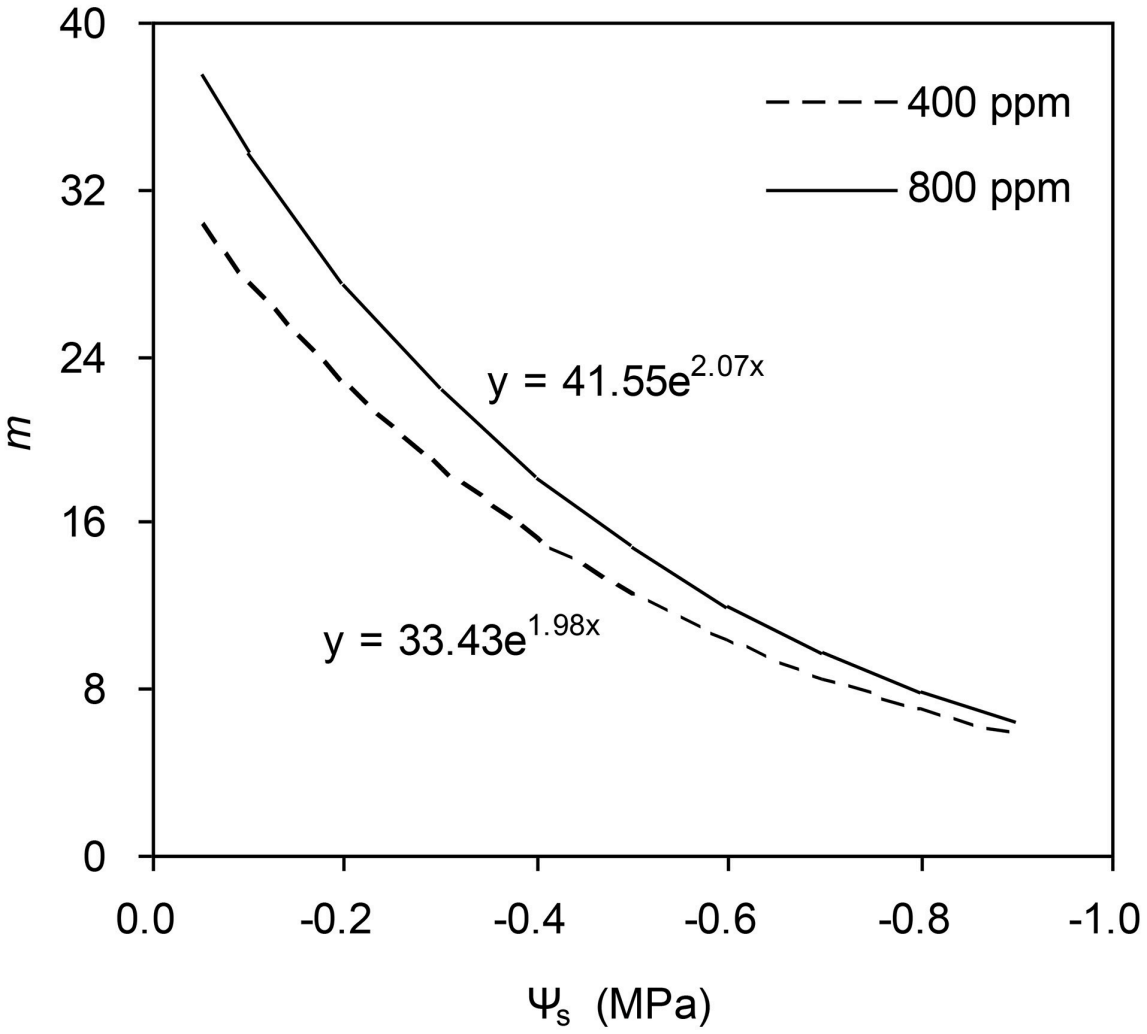
Simulation results of the effect of soil water deficits on the slope of the BB-model for a tomato leaf at [CO_2_] concentration of 400 and 800 ppm, respectively (details see Equation 2). Data is from experiment one (*n* = 25).

**Figure 4:**
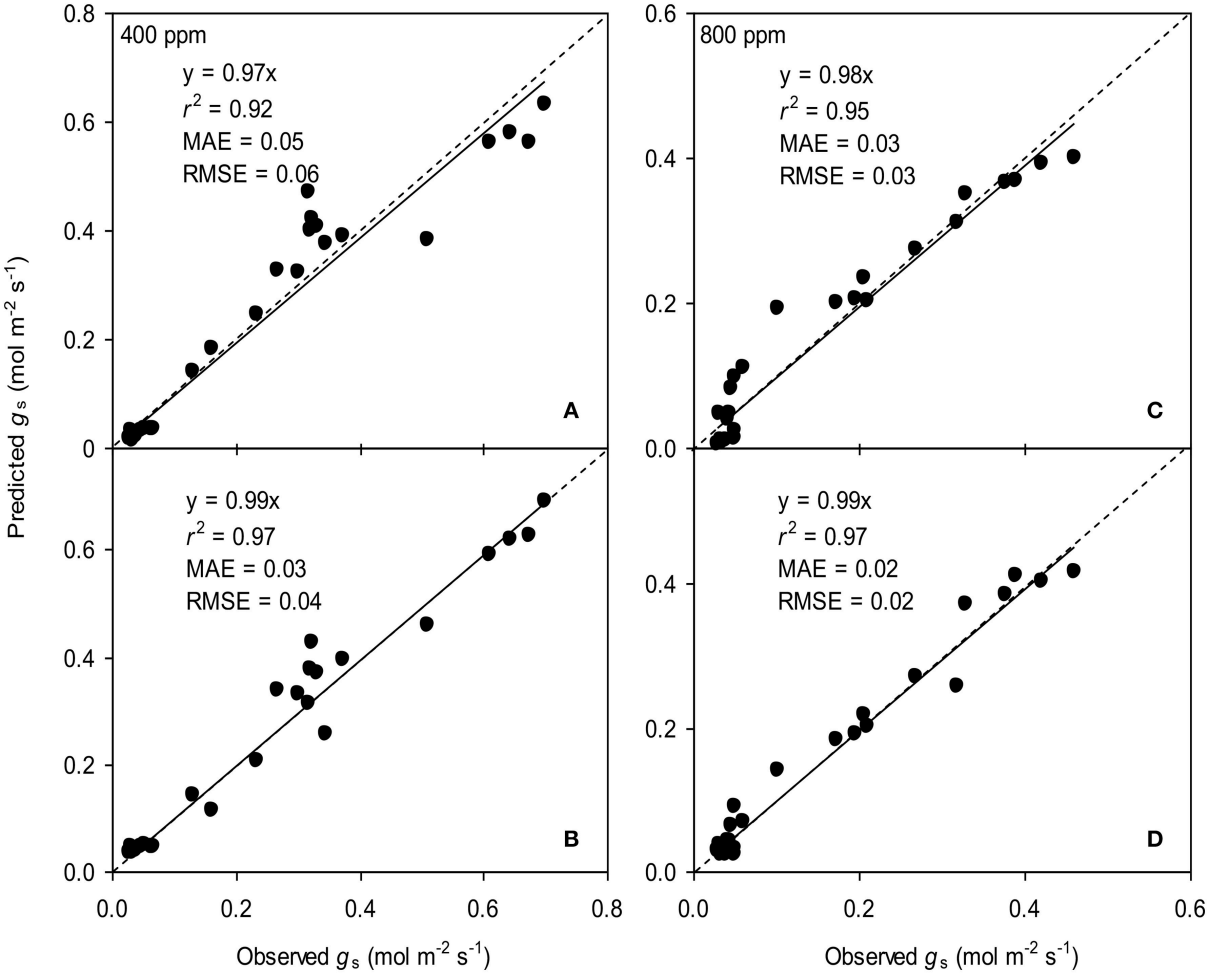
Observed and predicted stomatal conductance (*g*_s_) of tomato leaves predicted with the original **(A,C)** and the modified BB-model **(B,D**) at [CO_2_] concentration of 400 and 800 ppm, respectively. Data is from experiment one for model parameterization. The dashed lines indicate the 1:1 relationship.

**Figure 5:**
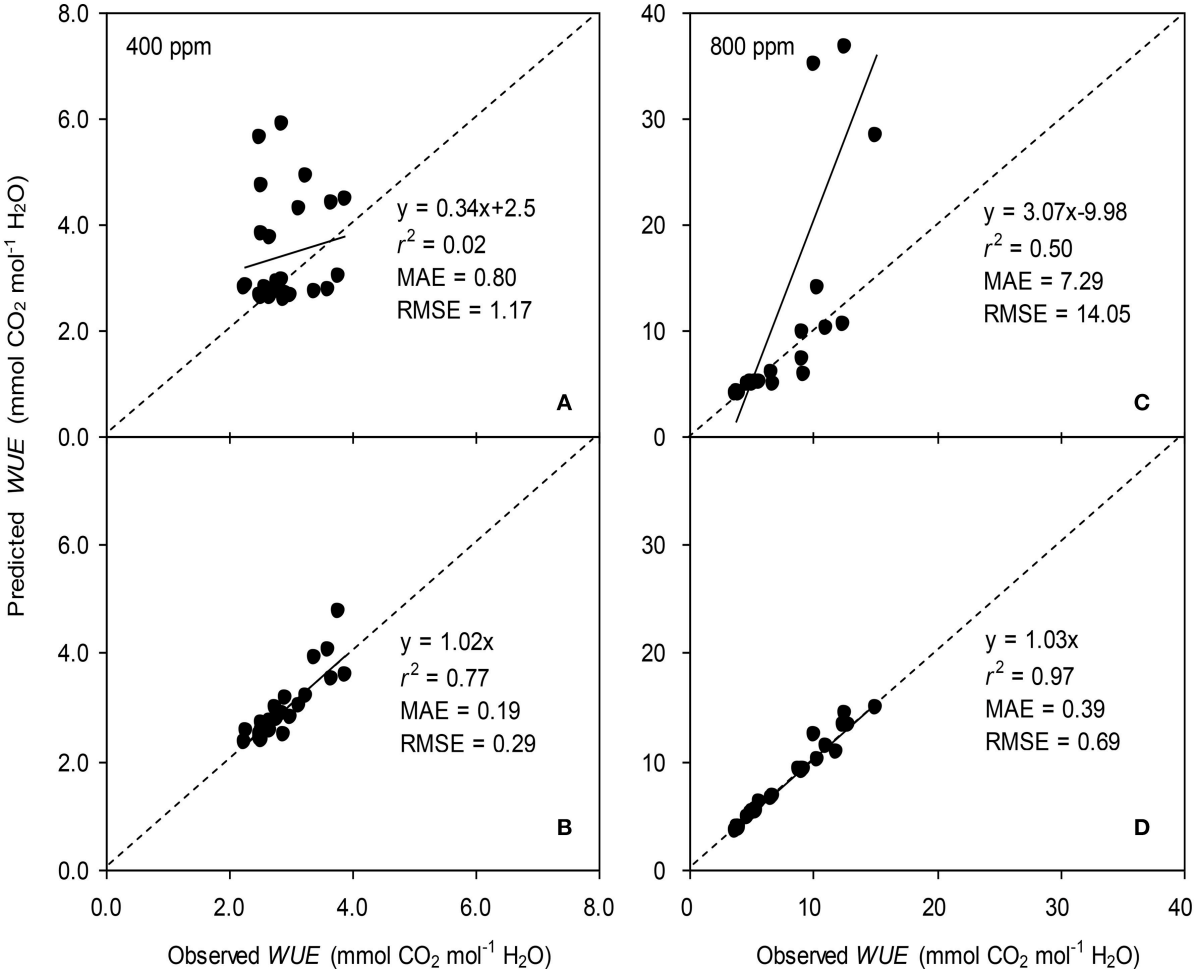
Observed and predicted water use efficiency (*WUE*) of tomato leaves predicted with the original **(A,C)** and the modified BB-model **(B,D)** at [CO_2_] concentration of 400 and 800 ppm, respectively. Data is from experiment one for model parameterization. The dashed lines indicate the 1:1 relationship.

To calculate the slope (*m*) of the BB-model, we first calculated *h*_s_ by using the observed *g*_s_ values. The Ball-index, viz. *P*_n_*h*_s_/*C*_s_ was then computed. By plotting the observed *g*_s_ against the Ball-index, an exponential, rather than a linear relationship between the two variables was found in both *a*[CO_2_] and *e*[CO_2_] (400 and 800 ppm) environment (Figure 2). Thus, *m* was not a constant indicating that *g*_s_ is not linearly correlated with *P*_n_ and *h*_s_. Accordingly, the effects of soil water deficits on *m* (i.e., Equation 2) must be taken into account. This was done by incorporating Equation (2) into Equation (9). The simulation results show that the initial slope of the BB-model (i.e., *m*_i_) was 33.43 and 41.55 at *a*[CO_2_] and *e*[CO_2_], respectively; while the both of actual slope (*m*) decreased exponentially with declining Ψ_s_ (Figure 3; Table 1). Also, the *m*_*i*_ was significantly greater for plants grown under *e*[CO_2_] than under *a*[CO_2_]. From Figures 4, 5 it can be seen that the modified BB-model significantly improved the *g*_s_ and *WUE* simulations as compared with the original BB-model due to the higher *r*^2^, lower MAE and RMSE values in both *a*[CO_2_] and *e*[CO_2_] environment.

**Table 1:** Parameters of the original and modified BB-models at [CO_2_] concentration of 400 and 800 ppm obtained from the multi-regression (Equation 9) over the data of experiment one.

**Treatment**	**Model**	**Slope (*m* or *m*_*i*_)**	**Intercept (*g*_0_) (mol m^−2^ s^−1^)**	**β (MPa^−1^)**
400 ppm	Original BB-model	26.85 (23.53 ~ 30.17)	−0.024 (−0.069 ~ 0.021)	Not relevant
	Modified BB-model	33.43 (29.57 ~ 37.29)	0.019 (−0.013 ~ 0.051)	−1.98 (−2.86 ~−1.10)
800 ppm	Original BB-model	37.62 (33.96 ~ 41.29)	−0.085 (−0.112 ~−0.057)	Not relevant
	Modified BB-model	41.55 (37.02 ~ 46.07)	−0.004 (−0.035 ~ 0.026)	−2.07 (−3.21 ~−0.93)

*The original BB-model has a constant slope, m; while the modified BB-model has an initial slope, m_i_ and which is reduced exponentially by soil water deficits (Equation 2). Values in the parentheses are 95% confidence intervals of the parameters (n = 25)*.

### Model validation

**Figure 6:**
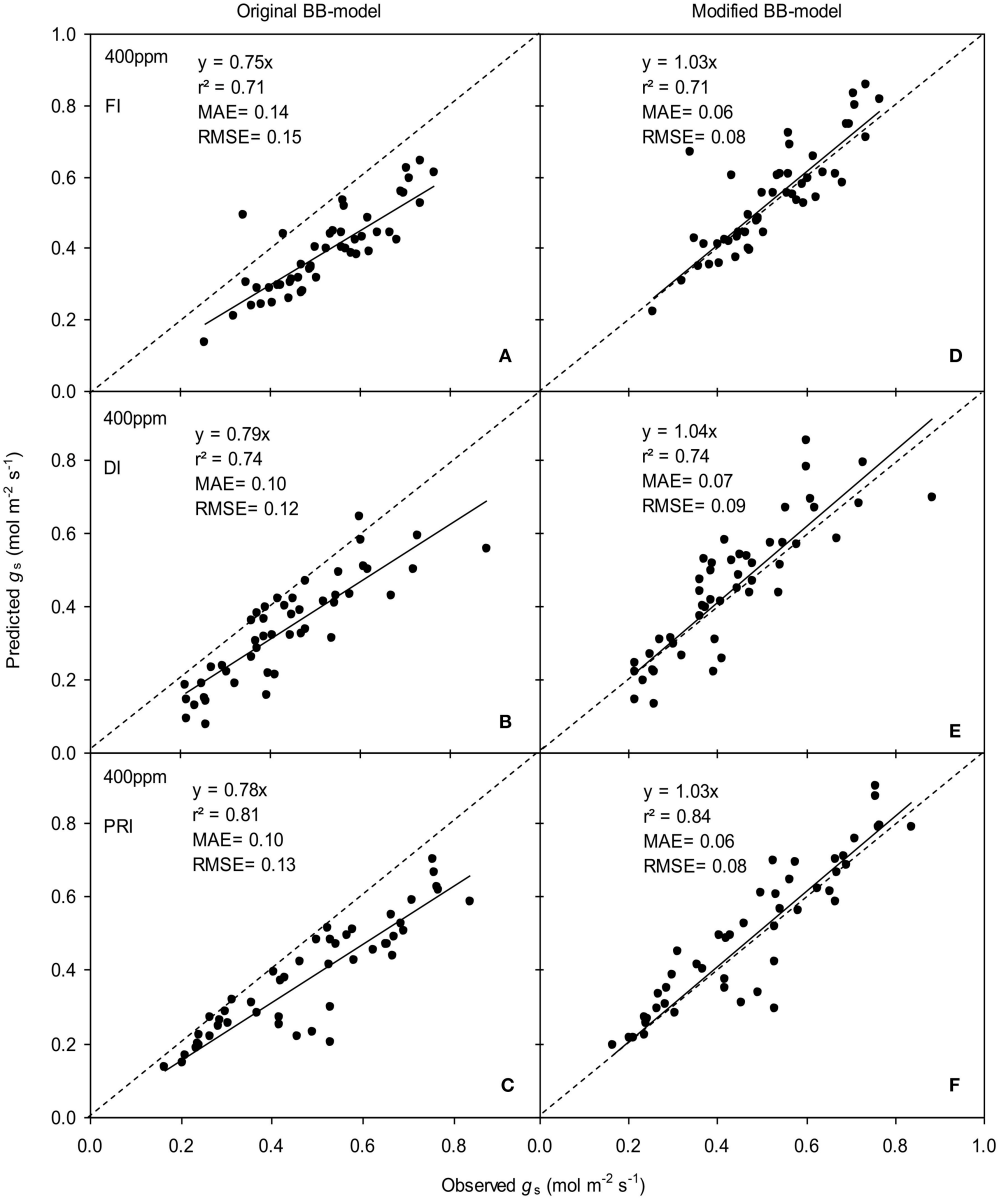
Observed and predicted stomatal conductance (*g*_s_) of experiment two data with the original **(A–C)** and the modified BB-model (**D–F)** of a tomato leaf under full irrigation (FI) **(A,D)** (*n* = 48), deficit irrigation (DI) **(B,E)** (*n* = 48) and alternative partial root-zone irrigation (PRI) **(C,F)** (*n* = 48), treatments at [CO_2_] concentration of 400 ppm. The predictability of the models was compared by the coefficient of determination (*r*^2^), the mean absolute error (MAE), and the root mean square of error (RMSE) of the linear regressions between the observed and predicted *g*_s_. The dashed lines indicate the 1:1 relationship.

**Figure 7:**
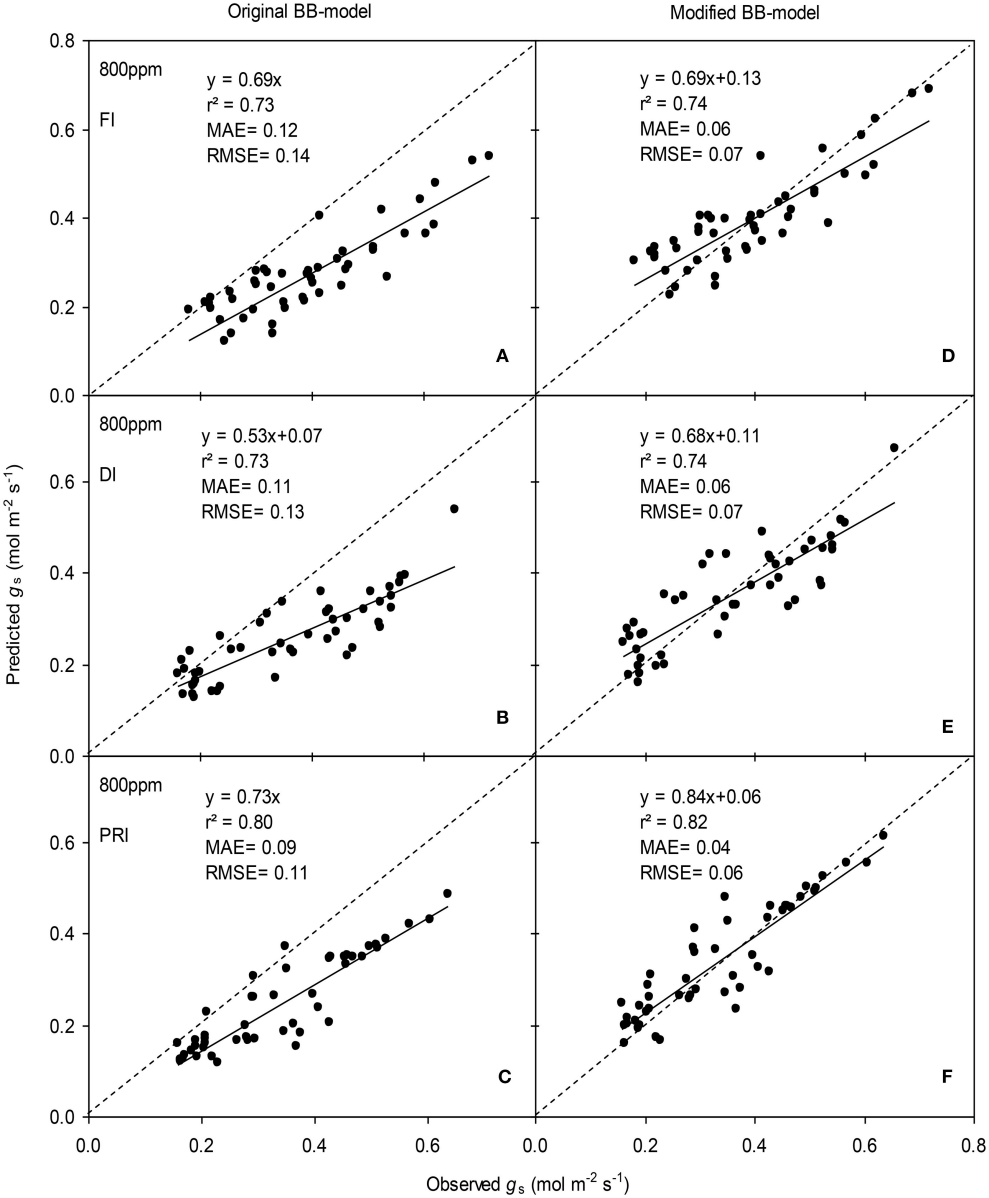
Observed and predicted stomatal conductance (*g*_s_) of experiment two data with the original **(A–C)** and the modified BB-model **(D–F)** of a tomato leaf under full irrigation (FI) **(A,D)** (*n* = 48), deficit irrigation (DI) **(B,E)** (*n* = 48) and alternative partial root-zone irrigation (PRI) **(C,F)** (*n* = 48), treatments at [CO_2_] concentration of 800 ppm. The predictability of the models was compared by the coefficient of determination (*r*^2^), the mean absolute error (MAE), and the root mean square of error (RMSE) of the linear regressions between the observed and predicted *g*_s_. The dashed lines indicate the 1:1 relationship.

The BB-models (with or without *m* modifications) were validated by the data obtained from the experiment II (Wei et al., unpublished). Shortly, for the FI plants Ψ_s_ was kept above −0.001 MPa; for the DI plants, Ψ_s_ ranged between −0.001 and −0.112 MPa; for the PRI plants, Ψ_s_ of the wet soil column was maintained above −0.001 MPa while that of the dry soil column ranged from −0.001 to −0.398 MPa during the treatment period. The model simulations indicated that both models were able to explain more than 71% of the variation in *g*_s_; for any FI, DI or PRI tomato plant, the modified BB-model was obviously superior to the original BB-model in predicting leaf *g*_s_ owing to its equal or higher *r*^2^, lower MAE and RMSE values at both *a*[CO_2_] and *e*[CO_2_] (Figures 6, 7).

**Figure 8:**
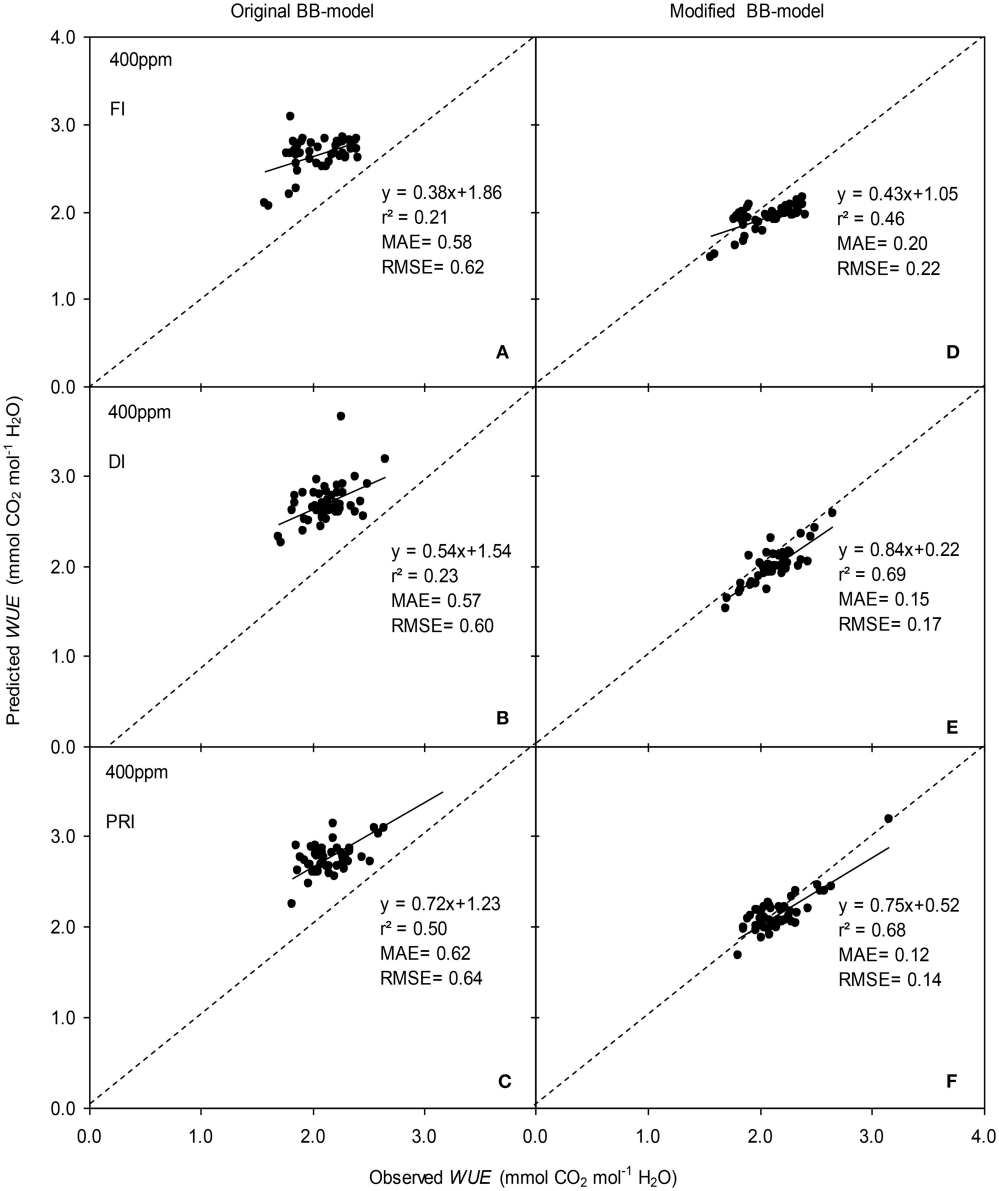
Observed and predicted water use efficiency (*WUE*) of experiment two data with the original **(A–C)** and the modified BB-model **(D–F)** of a tomato leaf under full irrigation (FI) **(A,D)** (*n* = 48), deficit irrigation (DI) **(B,E)** (*n* = 48) and alternative partial root-zone irrigation (PRI) **(C,F)** (*n* = 48), treatments at [CO_2_] concentration of 400 ppm. The predictability of the models was compared by the coefficient of determination (*r*^2^), the mean absolute error (MAE), and the root mean square of error (RMSE) of the linear regressions between the observed and predicted *g*_s_. The dashed lines indicate the 1:1 relationship.

**Figure 9:**
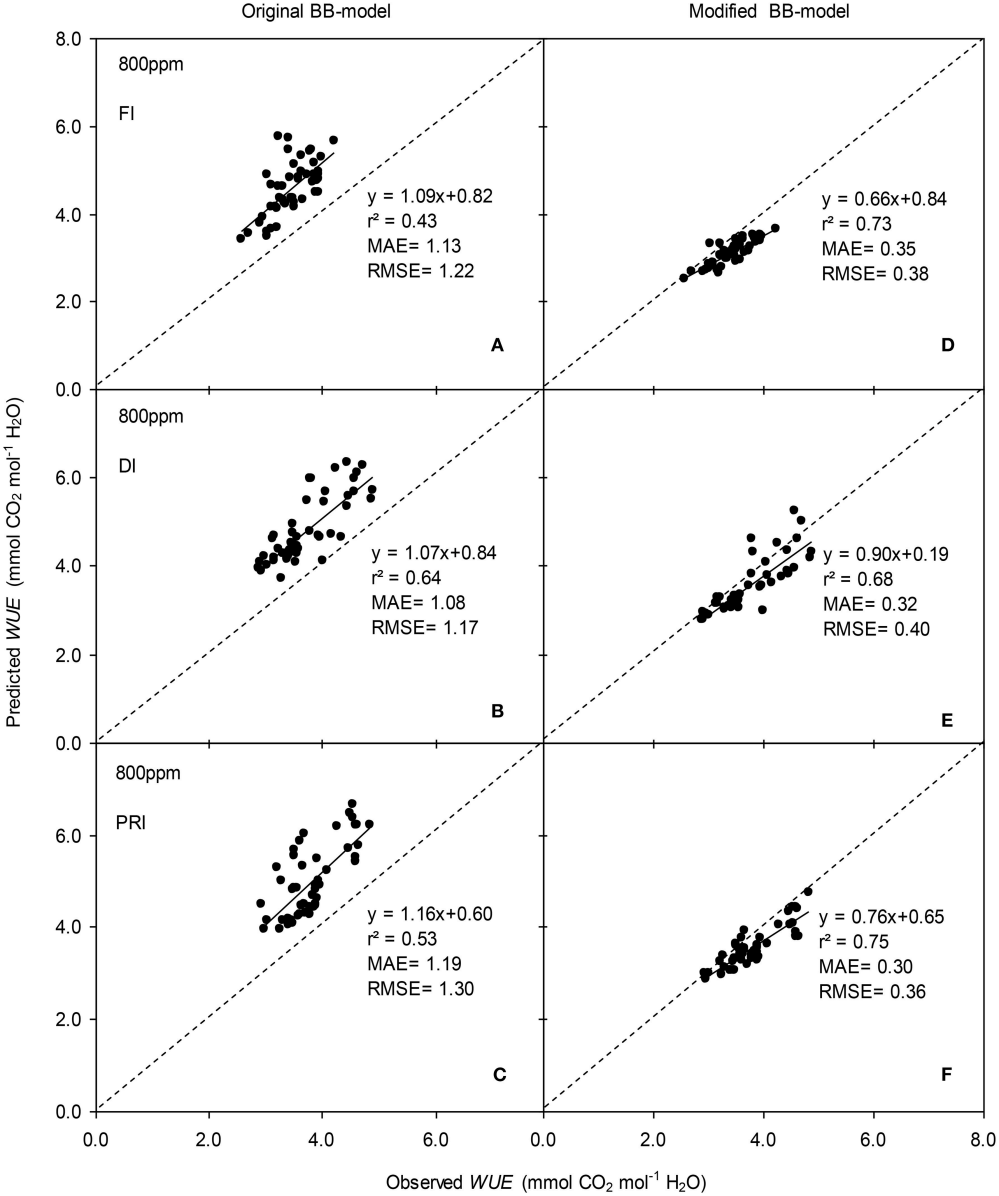
Observed and predicted water use efficiency (*WUE*) of experiment two data with the original **(A–C)** and the modified BB-model **(D–F)** of a tomato leaf under full irrigation (FI) **(A,D)** (*n* = 48), deficit irrigation (DI) **(B,E)** (*n* = 48) and alternative partial root-zone irrigation (PRI) **(C,F)** (*n* = 48), treatments at [CO_2_] concentration of 800 ppm. The predictability of the models was compared by the coefficient of determination (*r*^2^), the mean absolute error (MAE), and the root mean square of error (RMSE) of the linear regressions between the observed and predicted *g*_s_. The dashed lines indicate the 1:1 relationship.

**Figure 10:**
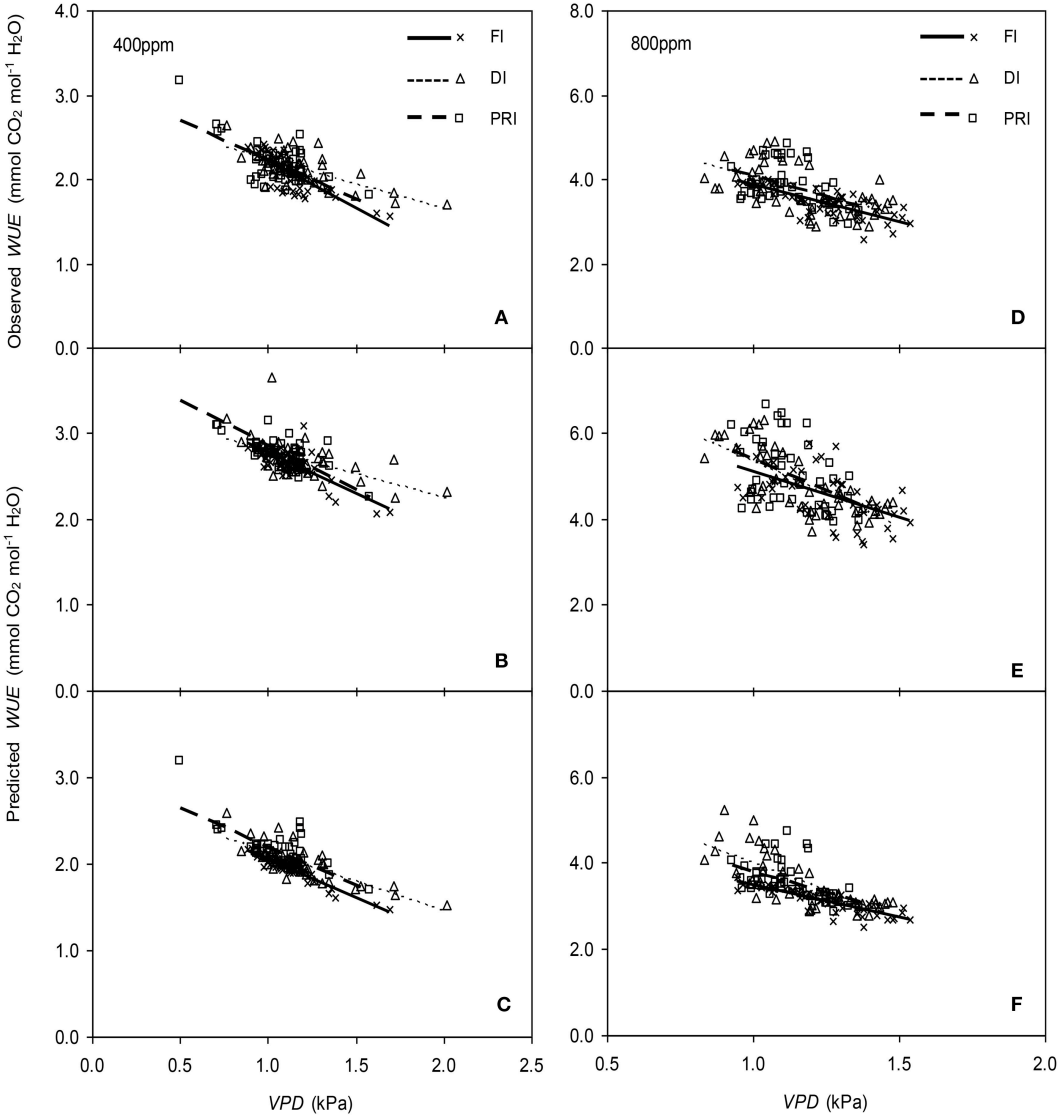
Relationships between the vapor pressure deficit (*VPD*) of the atmosphere and the measured photosynthetic water use efficiency (*WUE*) **(A,D)**, and the simulated *WUE* by the original **(B,E)**, and the modified BB-model **(C,F)** of tomato leaves under full irrigation (FI), deficit irrigation (DI) and alternative partial root-zone irrigation (PRI) regimes at [CO_2_] concentration of 400 and 800 ppm, respectively. The Comparison of regression lines under different irrigation treatments are seen in Table 2.

The original model showed poor predictability for *WUE* (0.21 < *r*^2^ < 0.64) of tomato leaves (Figures 8, 9). The modified BB-model performed better in predicting leaf *WUE* than did the original BB-model for any FI, DI or PRI tomato plant owing to its greater *r*^2^ (0.46 < *r*^2^ < 0.75), lower MAE and RMSE values in both *a*[CO_2_] and *e*[CO_2_] conditions. Figure 10 shows there was a negative relationship between *VPD* and *WUE*. The correlation between predicted *VPD* and *WUE* was not different among the FI, DI, and PRI treatments under the same BB-model and [CO_2_] environment (Table 2).

**Table 2:** Comparison of the regression lines between vapor pressure deficit of the air (*VPD*) and the predicted *WUE* under different irrigation treatments at [CO_2_] concentration of 400 and 800 ppm, respectively (Figure 10).

**Treatment**		**Observed**			**Predicted by BB-model**			**Predicted by modified BB-model**		
		**Slope**	**Y-intercept**	***r*^2^**	**Slope**	**Y-intercept**	***r*^2^**	**Slope**	**Y-intercept**	***r*^2^**
400 ppm	FI	−1.11 ± 0.13	3.32 ± 0.15a	0.77^***^	−0.93 ± 0.11	3.71 ± 0.13a	0.78^***^	−0.86 ± 0.04	2.91 ± 0.05a	0.96^***^
	DI	−0.58 ± 0.09	2.81 ± 0.11a	0.68^***^	−0.54 ± 0.12	3.34 ± 0.14a	0.56^***^	−0.68 ± 0.08	2.81 ± 0.09a	0.79^***^
	PRI	−0.92 ± 0.15	3.14 ± 0.16a	0.67^***^	−1.04 ± 0.14	3.89 ± 0.15a	0.75^***^	−0.90 ± 0.13	3.09 ± 0.14a	0.72^***^
800 ppm	FI	−1.74 ± 0.22	5.61 ± 0.27a	0.76^***^	−2.14 ± 0.46	7.23 ± 0.57a	0.57^***^	−1.52 ± 0.13	5.01 ± 0.17b	0.86^***^
	DI	−1.81 ± 0.37	5.85 ± 0.44a	0.58^***^	−3.03 ± 0.42	8.36 ± 0.50a	0.73^***^	−2.56 ± 0.33	6.52 ± 0.39a	0.76^***^
	PRI	−1.89 ± 0.56	5.96 ± 0.64a	0.44^**^	−2.92 ± 0.91	8.31 ± 1.03a	0.43^**^	−1.93 ± 0.47	5.73 ± 0.54ab	0.51^***^

Each value is represented as values ± SE (Standard Error). Different letters within each of the Y-intercept column indicate significant different between the regression lines of the treatments at P < 0.05 level (ANCOVA).

** and *** *indicate significance of the regression lines at P < 0.01 and < 0.001 levels, respectively*.

## Discussion

In the two pot experiments, the simulation of the BB-model in predicting *g*_s_ and *WUE* of tomato leaves grown in *a*[CO_2_] and *e*[CO_2_] environment under different irrigation regimes was performed. For both *a*[CO_2_] and *e*[CO_2_] plants, the original and modified BB-model could explain more than 71% of the observed variation in *g*_s_ for all irrigation regimes; the modified BB-model was notably superior to the original model in predicting *g*_s_ and *WUE*, although the two models showed relatively poor simulation in *WUE* of tomato leaves (*r*^2^ < 0.75) under different irrigation treatments.

It is well-known that partial stomatal closure leading to decrease in leaf *g*_s_ during progressive soil drying was mainly induced by the increased root-to-shoot chemical signaling (ABA) in moderate soil moisture deficit (Liu et al., 2005). Moreover, changes in environmental conditions especially *h*_*s*_ or *VPD* might mediate ABA action and influence leaf gas exchange (Wilkinson and Davies, 2002). A high *h*_s_ or lowered *VPD* may decrease the delivery and concentration of ABA in the guard cells, resulting in minimal increase in *g*_s_ and carbon assimilation without greater increase in transpiration (Speirs et al., 2013). In the current study, mostly the parameters *e*_i_ and *e*_a_ in the BB-model could well characterize the alteration in *h*_s_ or *VPD* and modulation on ABA catabolism in relation to the growth environment of the plants. In addition, the modulation of CO_2_ concentration in combination with *VPD* and soil water deficits on the ABA signaling and its regulation on *g*_s_ has not been well illustrated and needs further investigations (Yan et al., 2017).

A better understanding of whether there is physiological acclimation of *g*_s_ to water stress and *e*[CO_2_] is crucial for describing plant responses using the BB-model (Miner et al., 2017). The influence of soil water stress on the slope (*m*) has been contradictory. Misson et al. (2004) measured 350% variation in *m* of ponderosa pine during the developing season; similarly, Héroult et al. (2013) found that two eucalyptus species had significant reductions in *m* under drought. However, Xu and Baldocchi (2003) showed that *m* remains relatively constant for blue oak even under severe water stress. Hence, disparate proposals have been suggested to account for the effect of water deficit on *m* of the BB-model (Buckley and Mott, 2013). To emphasize the significance of the root-to-shoot ABA signaling in regulating *g*_s_ during mild soil drying, an ABA-based module was incorporated into the original BB-model to modify the *m* (Gutschick and Simonneau, 2002; Bauerle et al., 2004). The common point of those proposals for *m* modification has been that the initial slope of the BB-model, i.e., *m*_i_ is scaled downward in response to progressive soil drying (Liu et al., 2009). In the present study, the modified *m* is related to soil water potential (Ψ_s_) and it was found that such modification remarkably improved the predictability of model especially for the drought stressed tomato leaves grown at either *a*[CO_2_] and *e*[CO_2_] environment (Figures 4, 5). Besides, the *m* obtained from the model parameterization is higher (Table 1) than that observed mean value in C3 crops (Miner et al., 2017).

In soybean and wheat, the *m* significantly decreased when grown at *e*[CO_2_] (Bunce, 2004; Tausz-Posch et al., 2013). However, no change of *m* was noticed in five tree species (Medlyn et al., 2001) and soybean grown in a long-term free-air CO_2_ enrichment (FACE) experiment (Leakey et al., 2006), suggesting that acclimation of *g*_s_ is mostly along with the photosynthetic acclimation, resulting in an unchanged *m* in *e*[CO_2_] condition (Ainsworth and Rogers, 2007; Gimeno et al., 2016). In contrast to those, here the *m* in *e*[CO_2_] was significantly greater than that in *a*[CO_2_] environment (Table 1), implying *g*_s_ was independently acclimated to *e*[CO_2_] from *P*_n_. A greater *m* indicates a higher sensitivity of stomata to the plant growth environment (such as radiation, humidity, soil water availability, and CO_2_ concentration). In this study, the intercept of the modified BB-model, i.e., *g*_0_ for tomato leaves was relatively lower than that obtained in an earlier potato study by Liu et al. (2009), and was not statistically significant from zero. A decreased *g*_0_ indicates a low stomatal conductance in the dark. It has been reported that at *e*[CO_2_] the simulated *g*_0_ could be lower, greater or no change as compared with that at *a*[CO_2_] (Medlyn et al., 2001; Bunce, 2004; Leakey et al., 2006). The BB-model not only requires environmental variables, also need the plant photosynthesis. Earlier studies have adopted the Farquhar's photosynthesis model (Farquhar et al., 1980) as an integrated module in the BB-model for simulating leaf *P*_n_ (Gutschick and Simonneau, 2002; Bauerle et al., 2004; Misson et al., 2004). However, such model is largely apt to a model of the correlation between *P*_n_ and *g*_s_ resulted from the interdependence of those two factors, and it is useless for interpreting the causality (Liu et al., 2009). Numerous reports have confirmed that, applying the observed *P*_n_ as an input variable, the BB-model could performed successfully in predicting *g*_s_ over different species (Misson et al., 2004; Liu et al., 2009). In addition, accompanied with decreased *g*_s_, leaf *P*_n_ would maintained or increased under PRI or *e*[CO_2_] (Pazzagli et al., 2016), which could complicate the simulation of *P*_n_ for plants grown under PRI strategy combined with *e*[CO_2_] environment. Thus, in this study, the measured leaf *P*_n_ was used to improve the model's performance in predicting *g*_s_ across a wide range of soil water status.

Figures 6, 7 illustrated that the modified BB-model performed better than the original BB-model in predicting leaf *g*_s_ of the three irrigation treatments at each atmospheric [CO_2_] concentration, especially for the PRI tomato plant with highest high *r*^2^, lowest MAE and RMSE values. It is evident that a decreased *g*_s_ of leaf grown at *e*[CO_2_] would affect plant water use and further contribute to the varied Ψ_s_ (Kaminski et al., 2014). Likewise, the soil water heterogeneity induced by the PRI treatment could markedly altered the root-to-shoot ABA signaling involved in regulating *g*_s_, and the physiological responses, including leaf *g*_s_ become more sensitive to the reduction of Ψ_s_ in the PRI than that in DI plant (Davies et al., 2002; Liu et al., 2008). Besides, previous study has revealed that xylem ABA concentration of PRI-treated potatoes is determined by Ψ_s_ and not by Ψ_s_-dry (Liu et al., 2008), consistent with the results that the model performed much better when using mean Ψ_s_ in the whole root zone than using Ψ_s_-dry to account for the effect of soil drying on *g*_s_ (Liu et al., 2009). Therefore, a modification of *m* using Ψ_s_ incorporating into the BB-model is desired and necessary for achieving a better prediction of PRI tomato *g*_s_ in the *e*[CO_2_] environment.

In the present study, similar to that for *g*_s_ simulation, the modified BB-model also showed a better predictability for tomato *WUE* at leaf scale of all irrigation regimes in both [CO_2_] concentration as compared to the original model, particularly for PRI-treated plant, although the improvement of the prediction was less significant (Figures 8, 9). This could be ascribed to the accumulated errors in several variables for calculating *WUE* from the modeled *g*_s_. Nonetheless, regardless of the Ψ_s_ effect, the original and modified BB-model normally could not distinguish the influence of irrigation regimes on *WUE* of tomato leaves in either [CO_2_] condition (Figure 10; Table 2), indicating a robust relationship between *WUE* and *VPD* among different soil moisture condition and atmospheric [CO_2_] concentration. It is well-known that PRI combined with *e*[CO_2_] plants could lead to an increase of *P*_n_, and a decrease of stomatal aperture, hence synergistically enhancing leaf *WUE* (Pazzagli et al., 2016). Thus, a greater *WUE* is expected for the PRI plant grown in *e*[CO_2_] environment as simulated by the modified *g*_s_ and BB-model.

Collectively, tomato *g*_s_ was independent acclimation to *e*[CO_2_] environment from *P*_n_. Introducing Ψ_s_ of whole root zone into the BB-model could improve the model predictability of the model on *g*_s_ and *WUE* of tomato leaves under combinations of different irrigation regimes and CO_2_ environments. This information is useful for better predicting *WUE* of tomato plant in a future drier and CO_2_ enriched environment.

## Author contributions

ZW: Done the experiment and finished the first manuscript; TD: The corresponding author, the work was supervised by him; XL: Help to do the experiment; LF: Help to do the experiment; FL: The corresponding author, the work was supervised by him.

### Conflict of interest statement

The authors declare that the research was conducted in the absence of any commercial or financial relationships that could be construed as a potential conflict of interest.
